# The Week's Work

**Published:** 1910-03-12

**Authors:** 


### The Week's Work.

### A HOSPITAL'S REVENUE AND ITS DEVELOPMENT.

The sustained support given to individual voluntary hospitals in the provinces by subscribers is much greater in volume than it is in London. Thus the Metropolitan General hospitals receive under 13 per cent. of their revenue from annual subscriptions, whilst the Provincial General hospitals obtain upwards of 27 per cent. from this source. In the same way the hospital revenue derived from Hospital Sunday, Hospital Saturday, and contributions from workpeople is much greater in the provinces than in London; the percentage being 6.3, 1.5, and .3 per cent. in the metropolis, compared with 7.4, 7.9, and 12 per cent. respectively in the provinces. In the case of donations, however, London receives 19 per cent. of its income from this source, compared with about 14 per cent. in the provinces. Of course, some allowance must be made for special circumstances which may affect the yield of contributions. Some energetic hospital managers have proved, however, that annual subscriptions can be gathered in considerable volume in the metropolis if sufficient brains and work are put into the business. Each hospital should have a well-organised department under the control of a Subscriptions Committee, which should be charged with the duty of raising each year sufficient revenue from voluntary sources to cover the expenditure. This plan is in force at Newcastle, where Sir Riley Lord has shown that the Newcastle Infirmary, by the work of its Subscriptions Committee, has increased the subscriptions by £14,000 in five years; the greater portion of the increase (£11,600) has come from the working classes, the contributions received from groups of working men, *i.e.* collieries, railway men, and so forth, have met the actual cost to the hospital entailed by the treatment of patients received from each section. The results so arrived at are remarkable, and reflect great credit upon all concerned. Thus the railway men, when they were informed that there was a deficiency of £500 on the year in their case, promptly subscribed the deficiency. The Durham miners, though there is still a deficiency, have increased their subscriptions in four years from £1,984 to £4,840. The Northumberland miners, who had a deficiency in the cost of patients of £1,000 a year, have now covered the whole cost of their patients. The results reported at Newcastle are most encouraging. It is remarkable, however, although the infirmary has existed for 161 years, that, out of a population of over 400,000 people, the names of only 439 persons appear on the annual subscription list. The truth is that the subscriptions of the working men at Newcastle are far and away ahead of all other classes of subscribers. We congratulate Sir Riley Lord on the good results achieved under his chairmanship and direction.

### MEN, MONEY, ORGANISATION, AND WORK.

When a voluntary hospital, especially in London, begins to languish financially it is usually a sign that the time has arrived when the governors should introduce new and young blood upon its committees. In these days a committee consisting of amiable, kindly, uninfluential people who have little or no idea of hospital work or the raising of funds for hospitals, however well-intentioned they may be, may in practice prove an incubus which no actively administered voluntary hospital can carry and prove financially successful. Every hospital needs an efficient and active chairman, with a few influential colleagues, at any rate, to give their time and personal services to the work with unfailing regularity and energy. There must also be a competent, trained, adequately remunerated, permanent staff. We take it that, speaking of them as a whole, the London hospitals were probably never better equipped in these respects than they are to-day. Exceptions to this happy condition can be readily recognised at this season of the year when annual meetings are being held and accounts presented. The annual meeting should be made an occasion when the energy of the more thoughtful governors might usefully expend itself upon a close study of the report and accounts followed by attendance at the meeting, in order to elicit reasons for diminished or inadequate revenue, for any apparent absence of strict economy, and generally to encourage the committee and executive by intelligent criticism and earnest purposeful suggestions. The utility of such action and personal service is not sufficiently recognised by hospital governors as a class. If it were not so, certain hospitals which are at present in want of men and more money would speedily be provided with both. There are vacancies for a few active chairmen at the present time; and the services of good men of business, prepared to give time and energy to hospital work would be welcomed if they would come forward as volunteers. We should be glad to help any such gentlemen, who care to give us their names confidentially, in their endeavour to find a congenial sphere of work in connection with some hospitals where an opening may exist at the moment.

### HOSPITAL STORES PURCHASED IN COMBINATION.

The discussion in the Question Box on a Central Store for Hospitals has created a good deal of practical interest. The chairmen and committees of hospitals in East London are considering the possibility of combining to obtain better treatment in purchasing drugs, provisions, and other commodities. Mr. Holland proposes that the London Hospital should put the requirements of the smaller hospitals in its own tender, and that the contractors should deliver direct to each hospital which joins the combination. At the present time those responsible for the purchase of stores at every welladministered hospital have to exercise judgment and to take every possible means to keep in touch with prices and markets. So each responsible official should obtain most valuable experience and learn his business. Each hospital buys in competition, and so prices, in this type of hospital, are kept down to the lowest possible point. Purchase in combination would tend to destroy the training of a large number of officials, would remove much of the present competition, and possibly, in the end, might prove more costly than the present system. Another point to be borne in mind is the plan on which the contractors carry out the supply of commodities to hospitals in London. There seems to be some understanding, implied or actual, between contractors, so that the firm who gets, say, the meat contract for one or two large hospitals in a given district usually secures most of the other hospitals therein. In this way the contractors are able to send out supplies of meat of varying quality to a given district each day, and there is reason to think that the keenest and most efficient officials invariably get the best of the supplies. If this be so, the less efficiently-administered institutions must suffer in regard to quality. Is it not likely that, in case of combination, the bigger institutions might get the advantage in quality, though the smaller ones may gain something in price? Any attempt of the kind will, of course, be experimental in its initial stages, and whatever decision may be come to we shall watch the results with great interest. So far as the provinces are concerned, we would direct attention to the point raised by a provincial matron in the Question Box this week. If, as is feared, the fact of a change of system in methods of supplies led to diminished interest and support from the residents in the district which a hospital serves, the results might be disastrous. We have never been in favour of accepting subscriptions to a charity from tradesmen who claimed in return a right to a share of the hospital's patronage, apart from quality and price. Hospitals should purchase the best goods in the most favourable market, for an opposite course might mean that an annual subscription from a local tradesman might cost the institution many pounds per annum.

### THE HOSPITAL FOR INVALID GENTLEWOMEN.

The new buildings erected at 19 Lisson Grove, to which this hospital will be transferred from 90 Harley Street, where it has done most valuable work for sixty-seven years, were opened by the Princess of Wales on the 7th instant. There is accommodation for thirty-two patients, and the L plan on which it is built, and all the arrangements, reflect great credit upon the architect, Mr. Claude Ferrier. The operation theatre is situated on the fourth story; below it are the quarters for the nurses, each of whom has a separate bedroom. The wards are situated on the first and second floors and consist of two wards with eight beds each and fourteen single-bedded wards. The lady superintendent's room and offices are on the ground floor. Miss Nightingale was superintendent of this institution during its first two years at 90 Harley Street, and she has taken a continuous interest in its welfare ever since. When the freedom of the City of London was presented to Miss Nightingale she requested that fifty guineas might be sent as a donation to this institution instead of being expended upon the usual elaborate box containing the freedom of the City. We wish that Miss Nightingale might be well enough to pay a visit to the new buildings, because we are confident she would be very much pleased with the arrangements, which are well thought out and planned, and include everything calculated to make this hospital most comfortable and efficient in every way. The management is to be congratulated upon the fact that it has been able to open the new hospital free of debt, thanks to an anonymous donation of £5,000 and to a substantial grant from King Edward's Hospital Fund. The hospital provides for numbers of a social class who, in their ordinary lives, are not often the objects of charity, but whose limited means render them unable to bear the heavy expenses entailed by long or serious illness. It is a hospital which was much wanted. Admission is by payment of fees ranging from a guinea to £2 10s. per week; but these fees only cover half the expense, the balance being made up by subscriptions and donations. We congratulate Mrs. Bridgeman, the lady president, Mr. Bridgeman, M.P., the honorary treasurer, and Mr. Hugh Parker, the honorary secretary, to whose commendable energy and zeal the provision of this invaluable addition to the sick accommodation of the Metropolis is largely due. The tablet commemorating the opening of the building by the Princess of Wales is most chaste. It bears the arms of the Princess of Wales in colours, and the design is one of the most attractive we have ever seen. A word of praise is due to the matron, Miss Houghton, whose work was so well done that the whole establishment, from top to bottom, was found in perfect order when we inspected it immediately before the opening ceremony.

### BOLTON INFIRMARY.

The report of Sir Henry Burdett on this institution, published in our issue of February 12, p. 574, seems to have given rise to much local comment. The Board of the Infirmary at a meeting under the presidency of Alderman W. Nicholson, J.P., held on the 3rd instant, resolved that the Council of the British Medical Association be requested to nominate an expert of the highest standing to report upon the hospital; that such expert be requested to visit the hospital and report fully and completely on its condition and administration, and to accompany his report with any practical recommendations likely to increase the efficiency of the hospital. We welcome this resolution as justifying the confidence expressed by Sir Henry Burdett that his report would be considered on its merits. Some misapprehension appears to have arisen in the local Press as to the criticisms about the children's wards. One of them is satisfactory, but two, that formerly used as a ward-kitchen and that known as the cot ward, were not so when inspected, for the reasons stated in the report we published on the 12th ultimo.

### THE DRUG MARKET.

Several alterations in quotations for drugs and chemicals have been made, most of them being in a downward direction. Quicksilver has been reduced in price, and in consequence makers of calomel, corrosive sublimate, and other salts of mercury have reduced their prices by one penny per pound. Other drugs which are slightly lower in value are jalapin, podophyllum resin, and sulphonal. Buchu leaves are now in better supply and prices still inclined to recede. Ipecacuanha, on the other hand, is again slightly dearer. Opium, which showed signs of becoming cheaper a few weeks ago, has become more settled and prices are, if anything, somewhat higher; should the demand become more active, there will probably be a further advance. Morphine is steady, and, so far as can be seen at present, there is little likelihood of any immediate reduction in price. Reports from Norway concerning the cod-fishing are more favourable; with the propitious weather which has prevailed the catch has improved, and as regards numbers is very little behind last year's catch at the same date; the yield of oil, however, is very considerably less than last season, and prices are materially higher than they were at the beginning of the season, although the advance which took place a fortnight or so back has been checked.

